# Fullerenol Eye Drops Mitigate UVB-Induced Cataract Progression by Inhibiting Oxidative Stress and Cellular Senescence

**DOI:** 10.3390/antiox15010118

**Published:** 2026-01-16

**Authors:** Lele Zhang, Shuying Chen, Zihao Yu, Yuting Su, Jingyu Zhao, Lanlan Hu, Jinglong Tang, Mingliang Zhang

**Affiliations:** 1 Tianjin Key Laboratory of Retinal Functions and Diseases, Tianjin Branch of National Clinical Research Center for Ocular Disease, Eye Institute and School of Optometry, Tianjin Medical University Eye Hospital, Tianjin 300384, China; lelezhang@tmu.edu.cn (L.Z.);; 2 Department of Ophthalmology, Shaoxing People’s Hospital, Zhejiang University, Shaoxing 312000, China; 3 Department of Occupational and Environmental Health, School of Public Health, Qingdao University, Qingdao 266071, China

**Keywords:** cataracts, UVB, Fullerenol, antioxidant, senescence, oxidative stress, *P53*, *CDKN1A*

## Abstract

Cataracts remain the leading cause of blindness worldwide, and surgery is currently the only effective clinical treatment, as no pharmacological therapy has yet been validated. Here, we explore Fullerenol, a hydroxylated fullerene derivative formulated as eye drops, as a potential nanomedicine for delaying cataract onset and progression. In UVB-induced mouse cataract models, topical Fullerenol preserved the lens transparency and histological structure. In human lens epithelial cells, Fullerenol reduced the oxidative stress, restored the mitochondrial function, alleviated the DNA damage, and suppressed the cellular senescence. RNA sequencing and pathway enrichment analyses further indicated that Fullerenol modulated the oxidative stress- and senescence-associated signaling pathways, including *MAPK* and *TGF-β* cascades, while downregulating the *p53–CDKN1A (p21)* axis. These findings provide new evidence that Fullerenol can mitigate photo-oxidative damage and age-related cellular dysfunction, highlighting its promise as a non-invasive and clinically translatable nanomedicine strategy for cataract management.

## 1. Introduction

Cataracts are the leading cause of blindness worldwide. The lens primarily exerts its antioxidant effects through glutathione-dependent mechanisms. UVB exposure induces oxidative stress in lens cells, leading to decreased levels of GSH and increased levels of GSSG (oxidized glutathione) in the cell nucleus [1]. Oxidative stress causes oxidation, cross-linking, cleavage, and dehydrogenation of lens proteins, resulting in their aggregation, and ultimately, the formation of cataracts [2,3]. The current treatment is primarily limited to surgical intervention. Although modern cataract surgery is effective, it carries potential risks such as posterior capsule opacification and retinal detachment, and its availability may be restricted in many regions [4]. A new minimally invasive cataract surgery method, which utilizes endogenous stem cells to preserve the integrity of the lens capsule and associated LECs, promotes functional lens regeneration in animals and humans. However, this approach still results in postoperative inflammation, and it is specifically designed for congenital cataracts with no confirmed efficacy for age-related cataracts [5]. Consequently, safer and more accessible non-surgical approaches are urgently needed. Over the years, multiple pharmacological candidates have been explored, including Rapamycin and 3-MA for age-related cataracts [6], endoglucase inhibitors [7], and fructose-3-kinase inhibitors for diabetic cataracts [8]. Clinically related agents such as Lanosterol [9] and Pirenoxine [10] have also been investigated, but, so far, no effective eye-drop formulations have been validated for clinical use.

Accumulating evidence implicates oxidative stress and cellular senescence as central drivers of age-related cataract formation. Ultraviolet B (UVB) irradiation induces DNA and protein damage in the lens by generating reactive oxygen species (ROS), leading to loss of transparency [11]. Epidemiological studies have demonstrated a strong association between sunlight exposure, especially UVB, and cortical cataract incidence [12,13,14]. At the molecular level, dysfunction or mutation of genes such as *FYCO1* [15,16] and *Grx2* [17] accelerates lens opacification, while aberrant activation of *CDKN1A (p21)*, a downstream effector of *p53* signaling, promotes lens epithelial cell senescence [18]. Consistently, oxidative stress markers—including glutathione depletion, decreased superoxide dismutase (SOD) activity, and lipid peroxidation—are well-established hallmarks of cataract pathogenesis [19]. Although compounds such as Tanshinone IIA [20], Melatonin [21], and Cordyceps cicadae [22] have shown protective effects against oxidative injury in experimental models, no pharmacological agent has been clinically validated for cataract prevention or therapy. Given this mechanistic evidence, targeting oxidative stress- and senescence-related pathways, particularly those involving *p53–CDKN1A* signaling, represents a rational strategy for intervention. In this context, the potent antioxidant and anti-senescence properties of Fullerenol may provide a promising approach for developing non-surgical therapies against cataracts.

Fullerenol, a water-soluble derivative of Fullerene, exhibits strong antioxidant properties and has been explored in diverse oxidative stress-related conditions, including cancer, cardiovascular injury, and intervertebral disk degeneration [23,24,25,26,27]. In the eye, recent studies have demonstrated that Fullerenol protects the cornea from UVB-induced oxidative injury by suppressing ROS accumulation and preserving mitochondrial function [28], and rescues light-induced retinal damage by modulating Müller glia cell fate through *Nrf2/Wnt10a/TGF-β* signaling [29]. These findings highlight its therapeutic relevance for ocular diseases associated with photo-oxidative stress. At the cellular level, Fullerenol interacts with lipid membranes through van der Waals and dipole interactions [30,31], with the uptake mediated by endocytosis and clearance via exocytosis [32,33,34]. It can also associate with mitochondrial membranes, and its binding is concentration-dependent [35,36]. Despite these advances, the antioxidant and anti-senescence potential of Fullerenol in the lens has not been established.

Given its ability to neutralize ROS and modulate senescence pathways, the primary aim of this study was to determine whether Fullerenol eye drops protect against UVB-induced lens injury in vivo and alleviate UVB-induced oxidative stress and aging in lens epithelial cells in vitro. In parallel, we sought to elucidate the molecular mechanisms underlying these protective effects, with particular emphasis on the *p53–CDKN1A* signaling pathway and its role in regulating oxidative stress-associated senescence and DNA damage responses.

## 2. Materials and Methods

### 2.1. Materials

Fullerenol (C_60_(OH)_22_) was synthesized and characterized based on previously established methods [37]. It was dissolved in sterile water to obtain an 8 mM stock solution, and working concentrations of 80 and 160 μM were used in subsequent experiments. Reduced glutathione (GSH, CAS NO. 70-18-8, Yeasen Biotechnology, Shanghai, China) was prepared as a 160 mM stock in sterile water, with a working concentration of 160 μM.

Fullerenol characterization was primarily conducted by observing the nanoparticle morphology via transmission electron microscopy (TEM) (Hitachi, Tokyo, Japan, HT7800), whilst the particle size and zeta potential were measured using a dynamic light scattering (DLS) analyzer (Malvern Zetasizer Nano ZS90UK, Malvern, UK).

For topical ocular administration, the Fullerenol eye-drop formulation had a measured pH of 7.2 ± 0.5, which is comparable with physiological tear fluid. All solutions were freshly prepared and sterilized using a 0.22 μm filter before use.

### 2.2. Animals and Housing

Six-week-old *C57BL/6J* mice were obtained from Charles River Laboratories (Beijing, China). Animals were housed under controlled conditions (22–25 °C, 55 ± 5% humidity, 12 h light/dark cycle) with free access to food and water. Animal care and experimental procedures were carried out according to the ARVO Guidelines for the Use of Animals in Ophthalmic and Vision Research and were approved by the Laboratory Animal Management Committee, Tianjin Medical University Eye Hospital on 30 December 2024 (Experimental Protocol Number: TJYY2024120263).

### 2.3. UVB-Induced Cataract Model and Treatment

Mice were randomized into four groups: control, UVB, UVB + GSH, and UVB + Fullerenol. As previously studied by the author, except for the controls, animals were exposed to 302 nm UVB light (LUYER, XX-15BL, 15 W) at 15 cm for 30 min daily over 12 days [16]. From day 7, GSH or Fullerenol eye drops (160 μM, once daily) were administered until the mice were euthanized. The lens opacity was assessed using slit-lamp microscopy at 0, 4, 7, 10, 12, and 14 days. On day 15, the lenses were collected for histological and molecular analyses. Cataract grading was performed by investigators blinded to the group allocation.

### 2.4. Histology and Immunohistochemistry

Lens tissues were fixed, paraffin-embedded, and sectioned for hematoxylin and eosin (H&E) staining or TUNEL immunofluorescence with DAPI nuclear counterstaining. Sections were imaged using fluorescence microscopy.

### 2.5. Cell Culture and Treatments

Human lens epithelial cells (HLE-B3) obtained from the laboratory of the co-author Dr. Shuying Chen were cultured and maintained following the same conditions and procedures as previously reported [16,38]. The cells were cultured in DMEM/F12 with 10% FBS and 1% penicillin–streptomycin. The cells were treated using UVB irradiation for 10 min (LUYER XX-15BL). According to the manufacturer’s specifications, the nominal irradiance output was approximately 1.62 mW/cm^2^ at 25 cm. After the irradiation, fresh complete medium containing Fullerenol or GSH was immediately added for subsequent assays.

Subsequent assays included viability (CCK-8), proliferation (Ki67), senescence (SA-β-gal), oxidative stress (DCFH-DA), lipid peroxidation (MDA assay), and mitochondrial membrane potential (JC-1).

### 2.6. RNA-Seq and Bioinformatic Analysis

As mentioned previously, lens tissue samples were collected on day 14 from mice subjected to UVB irradiation and treated with Fullerenol/GSH. Three biological replicates were analyzed per group. Subsequently, all samples were sent to BGI (Wuhan, China) for further RNA-seq detection and analysis using the BGISEQ-500 platform. Differentially expressed genes (DEGs) were identified based on a fold change ≥ 2 and an adjusted *p*-value < 0.05 (|log_2_FC| ≥ 1, q < 0.05) as thresholds. KEGG enrichment and Gene Set Enrichment Analysis (GSEA) were performed to analyze genes associated with the “cellular senescence pathway.” Data mining and graphical presentations, including KEGG, GSEA, and heatmaps, were conducted using BGI’s in-house data mining system called Dr. Tom (http://report.bgi.com, accessed on 10 February 2025).

### 2.7. Immunofluorescence Staining

Human lens epithelial cells (HLE-B3) were fixed with PFA for 30 min at 24 h after the UVB irradiation and Fullerenol or GSH treatment. The cells were blocked with Block solution prepared with 5% goat serum at room temperature for 30 min. Furthermore, γ-H2AX (Proteintech, Rosemont, IL, USA, 83307-2-RR), CDKN1A (Proteintech, 28248-1-AP), and Phospho-P53 (Proteintech, 28961-1-AP) were incubated at 4 °C for 24 h, then incubated with rabbit IgG (Abcam, Cambridge, UK, AB150017) at room temperature for 2 h at a ratio of 1:500. The tablets containing DAPI were sealed and the tissue fluorescence staining was observed with an electron fluorescence microscope.

### 2.8. Real-Time Quantitative Polymerase Chain Reaction (RT-qPCR)

Total RNA was extracted from HLE-B3 cells using a commercial RNA extraction kit (EZBioscience, B0004DP, Shanghai, China) according to the manufacturer’s protocol. The RNA concentration and purity were determined spectrophotometrically. Reverse transcription was performed using a 4× RT Master Mix (EZBioscience, A0010CGQ), and quantitative PCR was conducted using an SYBR Green Master Mix (EZBioscience, A0012-R1) on a LightCycler 480 system (Roche, Basel, Switzerland). The primer is produced by Shanghai Shenggong Biotechnology Co., Ltd. (Shanghai, China). The PCR conditions were as follows: 95 °C for 5 min, 95 °C for 10 s, and 60 °C for 30 s for 45 cycles, and then 40 °C, 30 s. Then, the dissolution curve and CT value of the samples were analyzed.

The primer sequences for mouse CDKN1A were as follows: forward 5′-CGTGGACAGTGAGCAGTTG-3′ and reverse 5′-CAGAGGAAGTACTGGGCCT-3′. The accession number for the mouse CDKN1A is (NM_007669.5).

### 2.9. Western Blot

Following the HLE-B3 cells treatment with Fullerenol or GSH and UVB irradiation (10 min), cells in 6-well plates were harvested 24 h later. Then, 100 µL of RIPA lysis buffer containing 2% protease inhibitor cocktail and 2% phosphatase inhibitor cocktail was added to each well. After standing for 1 min, cells were scraped from the wells using a cell scraper and transferred to 1.5 mL microcentrifuge tubes. The lysates were allowed to stand for 10 min, followed by vortexing. This cycle (standing for 10 min then vortexing) was repeated twice more (3 times total). The lysates were centrifuged at 14,000× *g* for 20 min at 4 °C. The protein-containing supernatant was collected. Protein quantification: The protein concentration was determined using a BCA assay kit. Protein denaturation: The protein samples were denatured by adding Yeasen’s 5× Reducing Protein Loading Buffer and heating at 95 °C for 5 min. SDS-PAGE and transfer: The denatured proteins were separated using electrophoresis on a 12.5% SDS-PAGE gel and subsequently transferred onto a PVDF membrane. The PVDF membrane was blocked using 1× RapidBlock^TM^ solution for 15 min. The membrane was incubated overnight at 4 °C with the following primary antibodies diluted in blocking buffer: *CDKN1A* (~21 kDa, 1:2000, Proteintech, Wuhan, China), *P53* (~53 kDa, 1:10,000, Proteintech, Wuhan, China), *p-p53* (~53 kDa, 1:2000, Proteintech, Wuhan, China), and *β-actin* (~42kDa, 1:5000, Proteintech, Wuhan, China). The accession number for human *CDKN1A* is (NM_000389.5). The accession number for human *P53* is (NM_000546.6). The accession number for human *CDKN1A* is (NM_000389.5). The accession number for human *P53* is (NM_000546.6). Secondary antibody incubation: The following day, the membrane was incubated with appropriate horseradish peroxidase (HRP)-conjugated secondary antibodies (diluted 1:5000) for 2 h at room temperature. Protein bands were visualized using an enhanced chemiluminescence (ECL) substrate and exposure to X-ray film or an imaging system. Band intensities were quantified using ImageJ (Version number: ImageJ 1.53t) software.

## 3. Results

### 3.1. Fullerenol Alleviates UVB-Induced Lens Opacification In Vivo

The physicochemical properties of the prepared Fullerenol aqueous solution were first characterized. Supplementary Figure S1A,B show that Fullerenol displayed a mean particle diameter of 120 ± 20 nm. The zeta potential analysis demonstrated that the freshly prepared formulation exhibited an average surface charge of −41.2 ± 4.61 mV (Supplementary Table S1). After 14 days of storage, a slight rightward shift in the zeta potential distribution was observed, while the mean value remained highly negative (−42.9 ± 8.3 mV), indicating good colloidal stability over time.

To determine an appropriate working concentration, Fullerenol was initially evaluated at three higher doses (80, 160, and 800 μM) in vitro. Among these, 160 μM showed no detectable cytotoxicity while retaining favorable biological activity (Supplementary Figure S2). Consistently, histological examination of retinal sections revealed no obvious structural abnormalities or signs of toxicity following ocular exposure to 160 μM Fullerenol, supporting its suitability for subsequent in vivo experiments.

**Figure 1:**
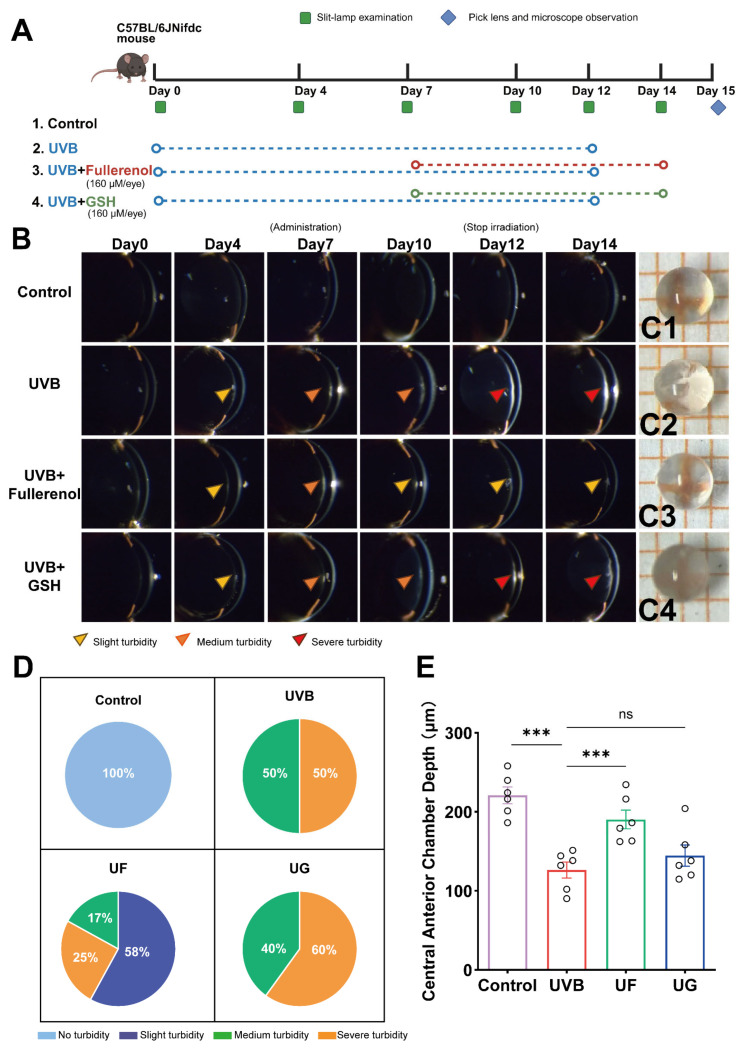
Protective effects of Fullerenol on UVB-induced lens injury in mice. (**A**) Experimental design showing the schedule of UVB irradiation, eye-drop administration (Fullerenol, 160 μM/eye; GSH, 160 μM/eye), and slit-lamp or microscopic examinations in *C57BL/6J* mice. (**B**) Representative slit-lamp images of lenses from each group (control, UVB, UVB + Fullerenol, UVB + GSH) on Days 0, 4, 7, 10, 12, and 14. Arrowheads indicate the degree of turbidity: yellow—slight, orange—moderate, red—severe. (**C1**–**C4**) Microscopic views of isolated lenses on Day 15. (**D**) Proportional distribution of lens turbidity grades in each group on Day 14 (*n* = 12 eyes per group). (**E**) Quantification of central anterior chamber depth on Day 14 (*n* = 6 eyes per group). Data are presented as mean ± SEM. Statistical significance was assessed using one-way ANOVA followed by Tukey’s post hoc test; ns: *p* > 0.05; *** *p* < 0.001.

To evaluate the therapeutic potential of Fullerenol against UVB-induced cataract formation, longitudinal slit-lamp examinations were conducted from Days 0 to 14 (Figure 1(A,C1–C4)). Lenses in the control group remained transparent throughout the study, whereas UVB-exposed mice developed progressive opacification, with mild turbidity at Day 4 and severe opacity by Day 12. Extracted lenses at Day 15 confirmed these findings (Figure 1(C1–C4)). Quantitative analysis of cataract grading on Day 14 and central anterior chamber depth further substantiated these observations (Figure 1D,E).

Intervention studies showed distinct therapeutic effects. Mice treated with 160 μM glutathione (GSH) or Fullerenol displayed only moderate turbidity by Day 7. Notably, Fullerenol-treated mice exhibited a significant reduction in opacification from Day 10 onward compared with the UVB group (*p* < 0.05), while the GSH treatment provided only partial improvement. By Day 14, Fullerenol markedly suppressed the cataract progression, indicating superior therapeutic efficacy over GSH. Apoptotic changes in the lens epithelium were further assessed using TUNEL staining. Supplementary Figure S3A,B show that UVB irradiation induced a marked increase in apoptotic signals. The Fullerenol treatment significantly reduced the TUNEL-positive fluorescence compared with the UVB group. Although GSH also decreased the apoptotic signals, the extent of reduction was less pronounced than that achieved with Fullerenol.

### 3.2. Histology Shows Structural Preservation of the Lens Using Fullerenol

**Figure 2:**
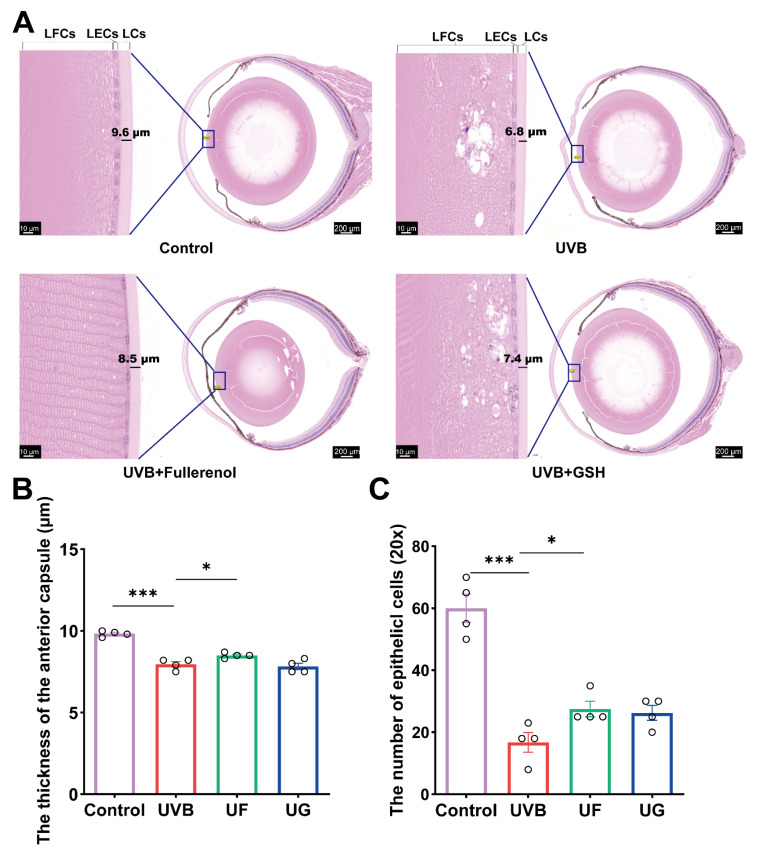
Histological assessment of UVB-induced lens injury and the protective effects of Fullerenol. (**A**) Representative H&E-stained sections of mouse lenses from each group (control, UVB, UVB + Fullerenol, UVB + GSH). Insets show higher-magnification images of the anterior lens capsule region, with the measured capsule thickness indicated. LFCs: lens fiber cells. (**B**) Quantification of anterior lens capsule thickness for each group (*n* = 4). (**C**) Quantification of lens epithelial cell (LEC) number per 20× field (*n* = 4 lenses per group). Data are presented as mean ± SEM from three independent experiments. Statistical analysis was performed using one-way ANOVA followed by Tukey’s post hoc test; * *p* < 0.05, *** *p* < 0.001.

Hematoxylin and eosin (H&E) staining was performed to assess the anterior capsule thickness and lens epithelial cell (LEC) density. UVB exposure markedly reduced the LEC numbers, thinned the anterior capsule, and caused severe cortical injury characterized via fiber disorganization and large vacuoles at the anterior pole (Figure 2A). Treatment with either GSH or Fullerenol reduced the size of the lens fiber vacuoles. Crucially, the Fullerenol treatment specifically resulted in thickening of the lens anterior capsule, a significant reduction in fiber cavities, and an increase in epithelial cell numbers. No additional ocular tissue abnormalities were observed following the Fullerenol administration (Figure 2A). Quantitative analysis confirmed that Fullerenol significantly improved the anterior capsule thickness and LEC counts compared with the UVB and GSH groups (Figure 2B,C; *p* < 0.05). In Supplementary Figure S3C, H&E staining of paraffin-embedded retinal sections show no apparent structural abnormalities following treatment with 160 μM Fullerenol, indicating that Fullerenol did not induce detectable retinal toxicity under the current experimental conditions.

### 3.3. Fullerenol Rescues Proliferation and Suppresses Senescence in UVB-Damaged HLE-B3 Cells

Given that Fullerenol significantly alleviated the lens opacity and preserved the tissue structure in vivo, we next investigated its protective effects at the cellular level using human lens epithelial HLE-B3 cells to further explore the underlying mechanisms.

**Figure 3:**
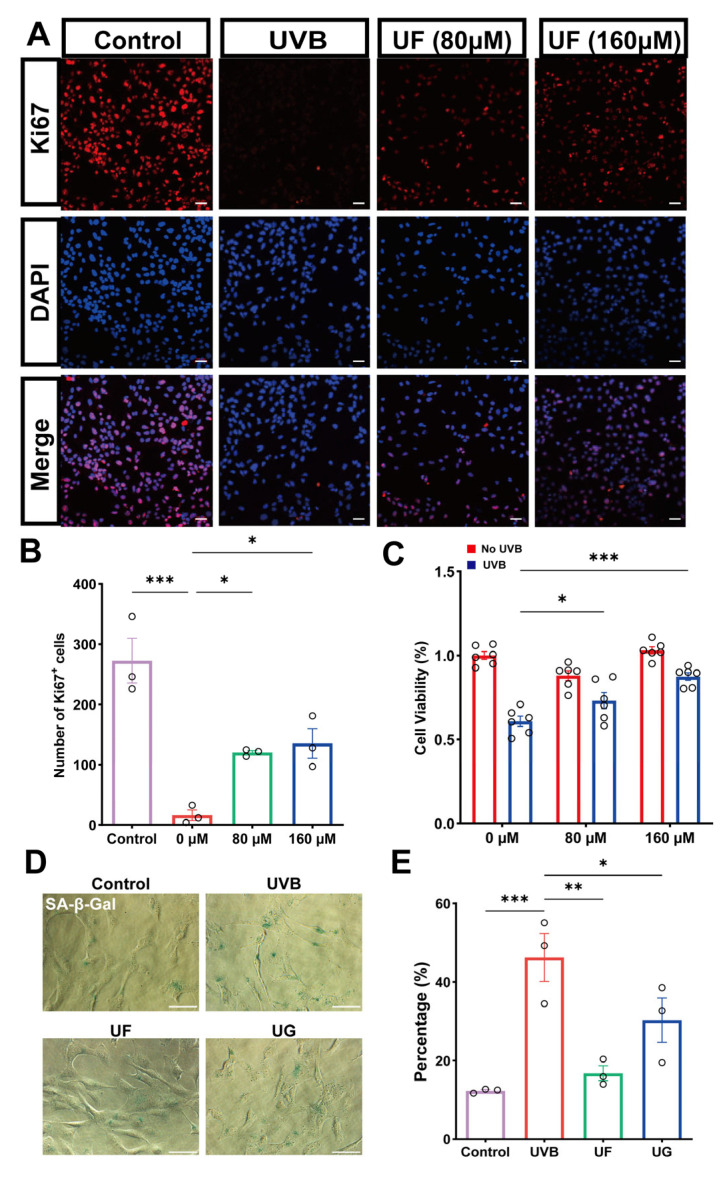
Fullerenol restores proliferation and viability while reducing senescence in UVB-damaged HLE-B3 cells. (**A**) Representative immunofluorescence images of Ki67 (red) and DAPI (blue) staining in HLE-B3 cells across groups (control, UVB, UVB + Fullerenol at 80 or 160 μM). Scale bar—50 μm. (**B**) Quantification of Ki67-positive cells (*n* = 3). (**C**) Cell viability measured by CCK-8 assay following UVB exposure and treatment with different concentrations of Fullerenol (*n* = 6). (**D**) Representative images of senescence-associated β-galactosidase (SA-β-gal) staining in HLE-B3 cells after UVB exposure and treatment with Fullerenol (UF) or GSH (UG). Scale bar—50 μm. (**E**) Quantification of SA-β-gal-positive cells across groups (*n* = 3). Data are presented as mean ± SEM from three independent experiments. Statistical significance was determined using one-way ANOVA followed by Tukey’s post hoc test; * *p* < 0.05, ** *p* < 0.01, *** *p* < 0.001.

Ki67 immunostaining revealed that UVB irradiation markedly suppressed the proliferative activity, whereas the treatment with 80 and 160 μM Fullerenol increased the number of Ki67-positive cells, indicating that Fullerenol can mitigate the inhibition of lens epithelial cell proliferation induced by UVB irradiation (Figure 3A,B). Consistently, CCK-8 assays showed a sharp decline in cell viability after UVB exposure, while Fullerenol (160 μM) significantly restored viability to ~85% of the control levels (Figure 3C).

Cellular senescence was further evaluated by senescence-associated β-galactosidase (SA-β-gal) staining. UVB irradiation induced a marked increase in SA-β-gal–positive cells, indicating the development of a senescent phenotype. Treatment with either GSH or Fullerenol significantly reduced the proportion of senescent cells, with a more pronounced effect observed in the Fullerenol-treated group (Figure 3D,E).

### 3.4. Fullerenol Attenuates Oxidative Stress in UVB-Irradiated HLE-B3 Cells

**Figure 4:**
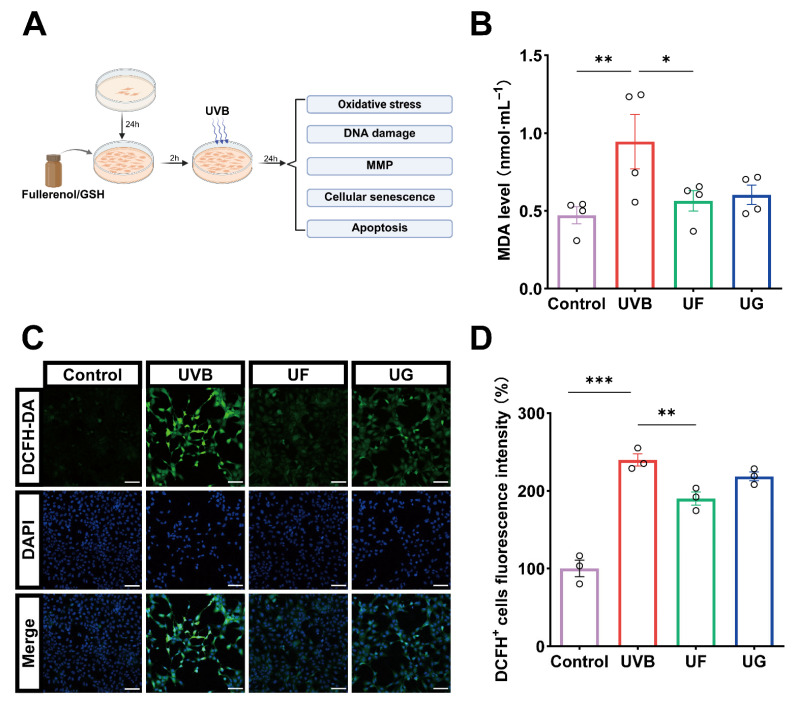
Fullerenol attenuates UVB-induced oxidative stress in lens epithelial cells. (**A**) Experimental workflow showing UVB irradiation and subsequent treatment with Fullerenol (UF) or glutathione (UG) in HLE-B3 cells. (**B**) Quantification of malondialdehyde (MDA) content across groups (*n* = 4). (**C**) Representative fluorescence images of intracellular ROS levels detected by DCFH-DA (green), with nuclei counterstained using Hoechst (blue). Scale bar—100 μm. (**D**) Quantification of DCFH-DA fluorescence intensity in each group (*n* = 3). Data are presented as mean ± SEM from three independent experiments. Statistical significance was determined using one-way ANOVA followed by Tukey’s post hoc test; * *p* < 0.05, ** *p* < 0.01, *** *p* < 0.001.

To clarify its restorative mechanisms, the oxidative stress markers, DNA damage, mitochondrial membrane potential (ΔΨm), cellular senescence, and apoptosis were assessed in HLE-B3 cells following the UVB irradiation and subsequent treatment with Fullerenol or GSH (Figure 4A).

As radiation damage is typically accompanied by oxidative stress, lipid peroxidation was first evaluated using an MDA assay, and intracellular ROS were quantified with a DCFH-DA fluorescent probe. The UVB exposure significantly increased lipid peroxide content compared with controls, whereas the Fullerenol treatment markedly reduced these levels (Figure 4B). Furthermore, intracellular reactive oxygen species (ROS) levels were determined using the DCFH-DA fluorescent probe. UVB irradiation induced a significant increase in fluorescence intensity in HLE-B3 cells relative to the control group (Figure 4C), indicating rapid ROS generation. The treatment with 160 μM Fullerenol markedly reduced the ROS levels. Notably, Fullerenol induced a more pronounced reduction in ROS levels than the GSH treatment. Quantitative fluorescence analysis further confirmed the significant intracellular ROS-scavenging activity of 160 μM Fullerenol (Figure 4D).

### 3.5. Fullerenol Mitigates DNA Damage and Mitochondrial Dysfunction Induced by UVB

We assessed the DNA damage and evaluated the mitochondrial membrane integrity and function using mitochondrial membrane potential (MMP) and γ-H2AX immunofluorescence staining detection, respectively.

**Figure 5:**
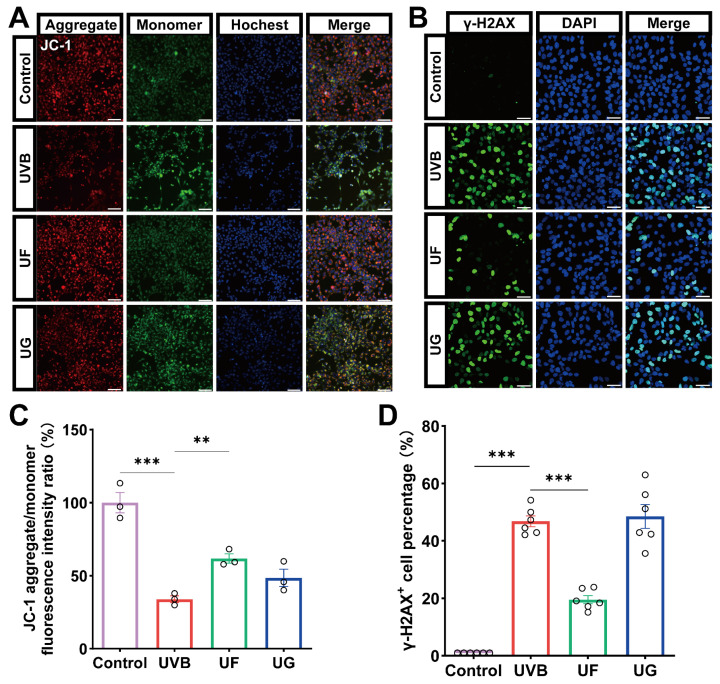
Fullerenol mitigates UVB-induced mitochondrial dysfunction and DNA damage in HLE-B3 cells. (**A**) Representative fluorescence images of mitochondrial membrane potential (MMP) changes detected using JC-1 staining. Red fluorescence indicates JC-1 aggregates, green fluorescence indicates JC-1 monomers, and nuclei were counterstained with Hoechst (blue). Scale bar—100 μm. (**B**) Representative immunofluorescence images showing DNA damage as γ-H2AX foci (green), with nuclei counterstained with DAPI (blue). Scale bar—50 μm. (**C**) Quantification of JC-1 aggregate/monomer fluorescence ratio across groups (*n* = 3). (**D**) Quantification of γ-H2AX^+^ cells across groups (*n* = 6). Statistical significance was determined using one-way ANOVA followed by Tukey’s post hoc test; ** *p* < 0.01, *** *p* < 0.001.

We assessed the cellular senescence by measuring changes in the mitochondrial membrane potential of the HLE-B3 cells. Under normal conditions, JC-1 within the mitochondria of HLE-B3 cells exists in its aggregated form, exhibiting bright red fluorescence with very weak green fluorescence. Upon UVB exposure, a sharp decline in JC-1 red fluorescence was detected, accompanied by an increase in green fluorescence in the cytoplasm, signifying a significant reduction in MMP. However, after treatment with Fullerenol, the red fluorescence increased while the green fluorescence diminished. In contrast, the HLE-B3 cells treated with GSH showed no significant change in MMP compared with the UVB group (Figure 5A,C). The alterations in JC-1 fluorescence patterns demonstrate that Fullerenol possesses a stronger capacity to reverse UVB-induced mitochondrial damage compared with GSH.

Intense γ-H2AX fluorescence in the UVB group demonstrated significant DNA damage in lens epithelial cells. Following the Fullerenol treatment, the fluorescence intensity markedly decreased (Figure 5B,D), indicating that Fullerenol effectively repairs UVB-induced DNA damage in HLE-B3 cells.

### 3.6. Transcriptomic Profiling Reveals Key Senescence- and Stress-Associated Pathways Targeted by Fullerenol

Given that oxidative stress, DNA damage, and mitochondrial dysfunction are tightly coupled with senescence signaling, RNA sequencing was performed to systematically characterize the transcriptional response to UVB irradiation and Fullerenol treatment. This genome-wide analysis revealed that several pathways related to cellular senescence and oxidative stress—including *MAPK* signaling, *TGF-β* signaling, and *CDKN1A* regulation—were significantly enriched, providing mechanistic insight into how Fullerenol mitigates UVB-induced lens epithelial injury.

**Figure 6:**
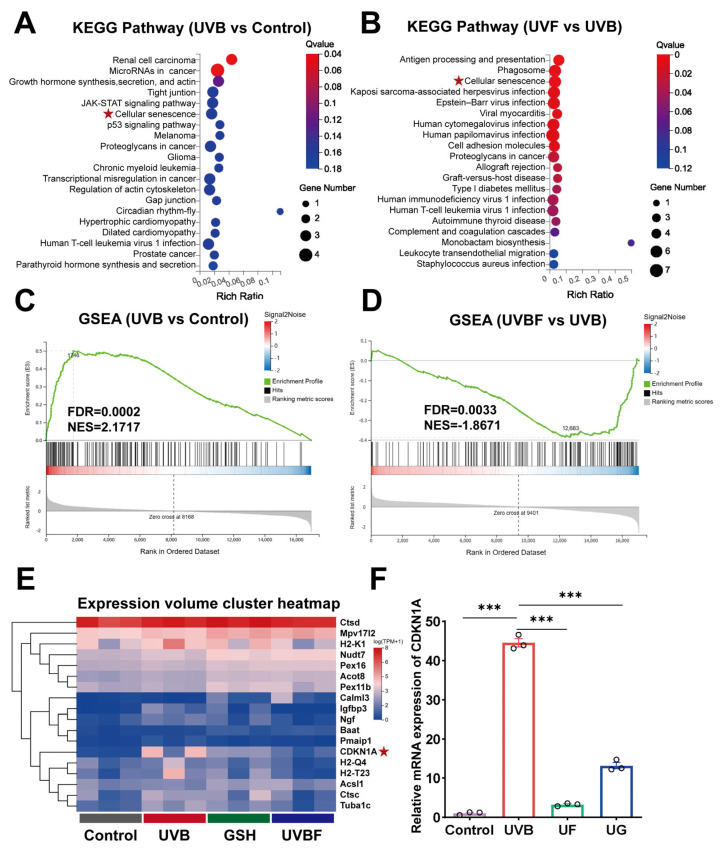
Transcriptomic profiling reveals senescence- and stress-related pathways modulated by Fullerenol. (**A**,**B**) KEGG enrichment analysis of differentially expressed genes (DEGs) in UVB vs. control and UVB + Fullerenol (UVF) vs. UVB. Cellular-senescence-related pathways were among the most significantly enriched categories. The red circle represents high expression. (**C**,**D**) Gene set enrichment analysis (GSEA) highlighting enrichment of senescence-related signaling pathways in UVB vs. control (**C**) and their suppression by the Fullerenol treatment (**D**). (**E**) Heatmap of representative senescence- and stress-related genes across groups. (**F**) Relative Cdkn1a mRNA expression validated by qPCR in mouse lenses (*n* = 3 per group). Data are presented as mean ± SEM. *** *p* < 0.001 by one-way ANOVA followed by Tukey’s post hoc test. The red stars in the figure indicates the signaling pathways/target genes validated in this study.

Figure 6 displays the distribution of differentially expressed genes (DEGs) and KEGG enrichment analysis across the experimental groups. This analysis revealed that cellular-senescence-related pathways (e.g., apoptosis/senescence signaling transduction) and oxidative stress-related pathways (e.g., *MAPK* signaling, *TGF-β* signaling pathway) were closely associated with cell proliferation (Figure 6A–D). We then selected 18 genes associated with oxidative stress and cellular senescence and generated a clustered heatmap, which further illustrated the differential expression patterns of genes within these pathways. The analysis revealed a marked upregulation of *CDKN1A* following UVB irradiation. Literature research indicates that *CDKN1A* is closely associated with cellular senescence and serves as a key senescence marker (Figure 6E). Subsequent qPCR validation across different experimental groups confirmed that UVB exposure significantly elevated the *CDKN1A* expression levels, whereas treatment with Fullerenol effectively suppressed this increase (Figure 6F).

### 3.7. Fullerenol Counteracts UVB-Induced Activation of the p53–CDKN1A Signaling Pathway

**Figure 7:**
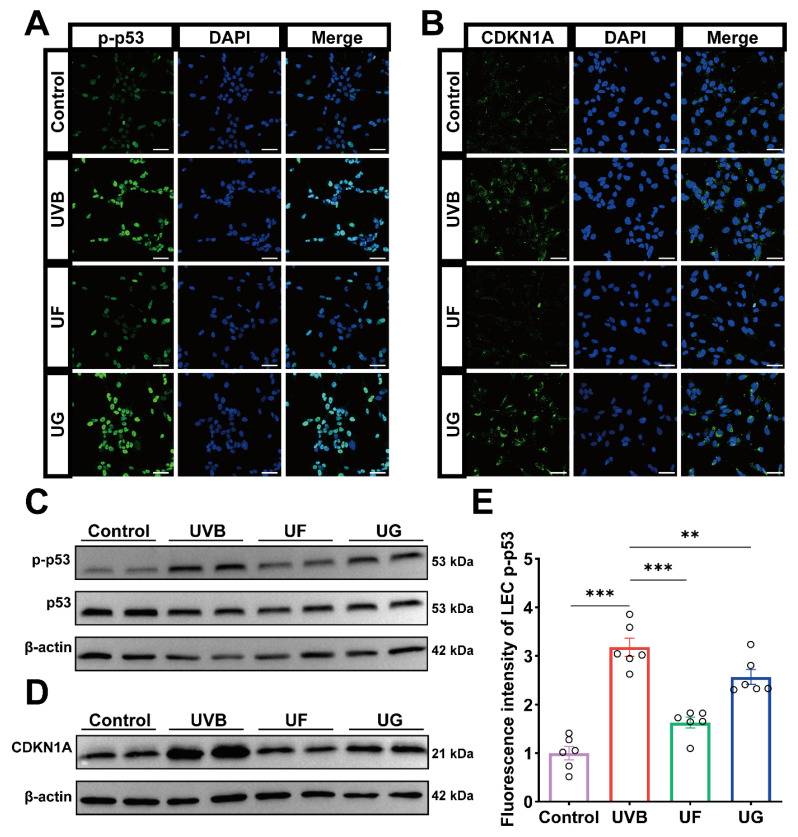

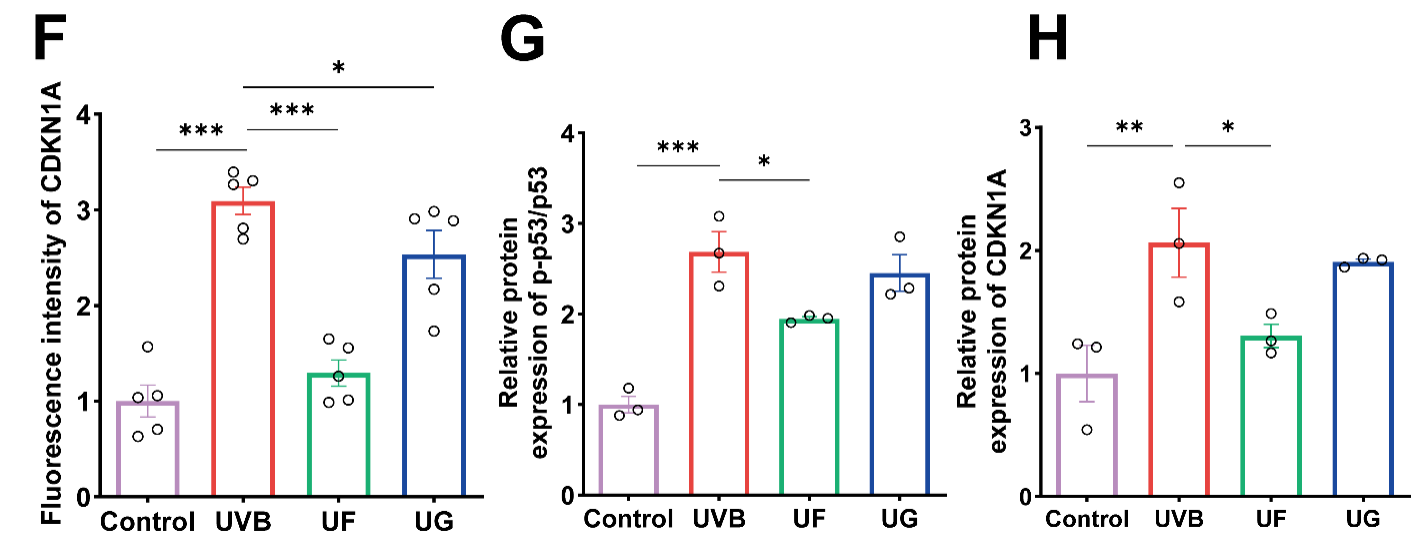
Fullerenol suppresses UVB-induced activation of the *p53–CDKN1A* signaling pathway in lens epithelial cells. (**A**) Representative immunofluorescence images of phosphorylated p53 (p-p53, green) with nuclei counterstained by DAPI (blue). Scale bar—50 μm. (**B**) Representative immunofluorescence images of *CDKN1A* (p21, green) with nuclei counterstained by DAPI (blue). Scale bar—50 μm. (**C**,**D**) Western blot analysis of *p53*, *p-p53*, and *CDKN1A* protein expressions across groups, with β-actin as the loading control. Protein sample volume 20 μg. (**E**,**F**) Quantification of fluorescence intensity for *p-p53* (**E**) and *CDKN1A* (**F**) in lens epithelial cells (*n* = 3). (**G**,**H**) Quantification of protein expression levels of p-p53/p53 (**G**) and *CDKN1A* (**H**) based on Western blot analysis (*n* = 3). Data are presented as mean ± SEM from three independent experiments. Statistical significance was determined using one-way ANOVA followed by Tukey’s post hoc test; * *p* < 0.05, ** *p* < 0.01, *** *p* < 0.001.

The RNA sequencing revealed that Fullerenol downregulates *CDKN1A* expression. *CDKN1A*, encoding the *p21* protein, is a critical cell cycle regulator and an established biomarker of cellular senescence. p53 is a well-established upstream regulator of *p21*. The *p53–p21* signaling pathway primarily modulates the cell cycle [38]. For this reason, we investigated whether the *p53–p21* pathway was affected by UVB irradiation and Fullerenol treatment in human lens epithelial cells. Immunofluorescence staining confirmed that UVB exposure markedly increased *phosphorylated p53 (p-p53)* and *CDKN1A* expression in HLE-B3 cells (Figure 7A,B), consistent with activation of the *p53–p21* senescence axis (Figure 7A,B). Quantitative analysis of the fluorescence intensity further substantiated these results (Figure 7E,F). Western blotting corroborated these findings, demonstrating that Fullerenol markedly decreased the *p-p53/p53* ratio and *CDKN1A* protein levels relative to the UVB-exposed controls (Figure 7C,D,G,H).

Collectively, these results suggest that Fullerenol attenuates HLE-B3 cellular senescence by modulating the *p53–CDKN1A* signaling pathway.

## 4. Discussion

Oxidative stress caused by ultraviolet radiation and endogenous metabolic processes is considered to play an important role in the pathogenesis of cataracts [39]. UVB irradiation can induce the production of active oxygen (ROS) and apoptosis in human lens epithelial cells (HLECs), leading to the formation of cataracts [40]. Consistent with this framework, our in vivo and in vitro data collectively support that Fullerenol—a hydroxylated, water-soluble Fullerene derivative—attenuates UVB-induced lens injury by reducing oxidative stress-associated cellular damage and suppressing senescence-associated phenotypes.

Fullerenol is a high-efficiency and stable free radical scavenger, which is easily soluble in water and has a stable structure. In addition, Fullerenol can protect mitochondrial proteins from oxidation, support the maintenance of mitochondrial membrane potential, and inhibit cell apoptosis induced by ionizing radiation, which has radiation protection properties [41,42,43,44]. Unlike traditional antioxidants and free radical scavengers, Fullerenol can quench free radicals through carbon addition reactions and hydrogen extraction reactions of functional groups.

Our findings indicate that Fullerenol improves the cell viability and proliferative capacity while reducing SA-β-gal positivity in UVB-damaged HLE-B3 cells, reflecting the attenuation of senescence-associated features. In parallel, Fullerenol reduces ROS accumulation and lipid peroxidation, restores mitochondrial membrane potential, and decreases γ-H2AX foci formation, supporting its role in mitigating oxidative stress-associated mitochondrial and genomic injury.

Transcriptomic analysis further revealed enrichment of stress- and senescence-related pathways following UVB exposure. Among these, the *p53–CDKN1A* axis was prioritized for validation because of its established role in UV-induced growth arrest and cellular senescence. Our RT-qPCR, immunofluorescence, and Western blot data consistently showed that Fullerenol suppresses *p53* phosphorylation and downregulates *CDKN1A* expression. As demonstrated in Figure 1 and Supplementary Figure S3A, mouse lenses exhibited opacity under slit-lamp examination 7 days after UVB irradiation. Paraffin section immunofluorescence staining 14 days post-irradiation revealed that the lens opacity was associated with apoptosis of epithelial cells induced by the UVB exposure. The HE staining results revealed that UVB irradiation thinned the lens capsule, while Fullerenol increased the capsule thickness and restored the epithelial cell numbers. We propose that these protective effects are closely associated with Fullerenol’s capacity to counteract oxidative stress and support proliferative homeostasis in lens epithelial cells. Although the present study did not fully delineate the molecular pathways linking antioxidant activity to tissue-level protection, the ability of Fullerenol to maintain lens transparency and delay cataract progression highlights its potential profound relevance in cataract prevention.

Several antioxidant or pharmacological strategies have been explored for cataract prevention, including Melatonin [45], Tanshinone IIA [20], Lanosterol [46], and Pirenoxine [10], with each targeting different aspects of cataract pathophysiology. Melatonin primarily exerts protective effects through its potent free-radical-scavenging capacity and mitochondrial protection, but Oral Melatonin demonstrates poor intraocular efficacy, while ophthalmic formulations remain immature [47]. Furthermore, as a hormone substance, its long-term use may induce adverse effects in humans [48]. Tanshinone IIA has demonstrated antioxidative and anti-apoptotic effects in experimental cataract models [20]; however, its relatively poor aqueous solubility poses formulation challenges for topical delivery [49]. Lanosterol has attracted attention for its potential role in reversing protein aggregation in the lens, yet Lanosterol exhibits poor water solubility and is prone to oxidation, necessitating complex formulations to produce eye drops [50]. Pirenoxine has been widely used clinically in some regions, but convincing evidence from large-scale randomized trials supporting its anti-cataract efficacy remains limited [10,51].

Compared with these candidates, Fullerenol offers several distinctive features. As a hydroxylated Fullerene derivative, it combines strong antioxidant capacity with high water solubility, enabling formulation as an aqueous eye drop. In contrast to small-molecule antioxidants that are rapidly consumed during redox reactions, Fullerenol can quench free radicals through multiple chemical mechanisms, potentially providing broader and more sustained protection against oxidative injury. Moreover, our data suggest that Fullerenol not only reduces oxidative stress but also attenuates senescence-associated signaling, particularly the *p53–CDKN1A* axis, linking redox regulation to cell-cycle control in lens epithelial cells. These properties may position Fullerenol use as a complementary strategy to existing antioxidant approaches rather than a direct replacement, especially in the context of oxidative stress-driven cataractogenesis.

Nevertheless, it should be emphasized that Fullerenol remains at the stage of fundamental research. Direct comparisons between these candidates are complicated by differences in models, dosing regimens, and endpoints. Future head-to-head studies using standardized cataract models and functional visual outcomes will be essential to define the relative advantages and limitations of Fullerenol in relation to established antioxidant and emerging nanomedicine strategies.

The Fullerenol used in this study is a hydroxylized Fullerene derivative with strong water solubility and stability [52], with a reported half-life of approximately 26 days [53]. Previous studies have suggested that Fullerene nanoparticles can cross cell membranes through both passive diffusion and active transport, exhibiting high permeability within lipid bilayers [54]. However, ocular drug delivery is inherently challenging due to physiological barriers such as tear dilution, nasolacrimal drainage, blinking, and the limited permeability of the cornea. Nevertheless, pharmacological studies indicate that small molecules can traverse the cornea, predominantly through passive diffusion [55]. In addition, drugs may cross static barriers, such as the cornea and conjunctiva, via transcellular or paracellular pathways: lipophilic compounds generally utilize the transcellular route, while hydrophilic agents preferentially pass through paracellular spaces [56]. Fullerenol differs from Fullerene in carrying numerous hydroxyl groups, rendering it an extremely hydrophilic nanomolecule. The observed protective effects may arise from limited anterior segment exposure, indirect modulation of the oxidative milieu, or partial penetration under injury-altered barrier conditions.

At present, isolating Fullerenol from intraocular tissues and precisely quantifying its concentration remain technically challenging, limiting pharmacokinetic characterization. Dedicated studies assessing Fullerenol levels in the tear film, cornea, aqueous humor, and lens tissue will therefore be essential to clarify its ocular disposition and exposure–response relationships.

Safety is another critical consideration. Previous studies on the biosafety of Fullerenol have yielded conflicting results. Some investigations suggest that concentrations up to 25 μM may induce cytotoxicity in certain cell types [57], whereas others have shown no toxicity, even at the dissolution threshold. For instance, in CHO-K1 cells, Fullerenol in the range of 12.4–249 μg/mL exhibited no significant genotoxicity [58]. In TC cells, cytotoxic effects were observed only when concentrations exceeded 10 μg/mL [59], while in HUVECs, even 200 μg/mL showed no obvious toxicity [59]. Similarly, in retinal studies, concentrations up to 500 μg/mL were well tolerated [29]. In a corneal UVB model, 25 μM Fullerenol applied topically also showed no toxicity [28].

Given that locally administered Fullerenol will inevitably be diluted and degraded before reaching the lens, in our study, in vitro toxicity testing demonstrated that 160 μM Fullerenol did not induce significant cytotoxicity in HLE-B3 cells while exerting pronounced protective effects. Discrepancies in reported toxicity across studies may arise from differences in solvents, dissolution methods, or cellular models [60].

Our histological assessment did not reveal overt retinal structural abnormalities after short-term topical administration. However, this represents only preliminary tolerability evidence. A comprehensive ocular safety evaluation—including irritation scoring, fluorescein staining, inflammatory markers, intraocular pressure monitoring, and long-term repeat-dose toxicology—remains indispensable before any translational conclusions can be drawn. Published reports on Fullerenol biosafety vary substantially between models and experimental conditions, underscoring the need for standardized safety profiling.

Several limitations of the present study should be acknowledged. First, the UVB irradiation protocol represents an acute injury model that does not fully recapitulate the chronic, multifactorial pathogenesis of age-related human cataract, which develops over decades through cumulative oxidative stress, metabolic alterations, and age-dependent proteostasis decline. Therefore, our findings should be interpreted as proof-of-concept evidence for oxidative-injury-driven cataract-like changes rather than direct clinical equivalence.

Second, cataract severity in this study was evaluated primarily using morphological and histological criteria. Functional visual outcomes, such as optokinetic response or visual acuity surrogates, were not assessed and should be incorporated in future work to establish true visual benefit.

Third, while transcriptomic profiling suggested involvement of multiple stress-related pathways (including *MAPK* and *TGF-β* signaling), we validated only the p53–*CDKN1A* axis. Broader mechanistic validation will be necessary to fully delineate the signaling networks modulated by Fullerenol.

Finally, although the short-term tolerability was acceptable under our experimental conditions, long-term ocular safety and systemic exposure remain unexplored. These aspects are critical for any future translational consideration.

Taken together, these limitations define a clear roadmap for future studies: integrating pharmacokinetic analysis, extended safety profiling, chronic-disease-relevant models, functional visual assessment, and deeper mechanistic validation will be essential steps toward evaluating the true therapeutic potential of Fullerenol in cataract prevention.

## 5. Conclusions

UVB irradiation induces lens opacification and visual impairment by triggering oxidative stress and cellular senescence in lens epithelial cells. In this study, we demonstrate that Fullerenol eye drops effectively attenuate UVB-induced oxidative damage, restore lens transparency in vivo, and protect lens epithelial cells in vitro. These findings provide compelling evidence that Fullerenol holds promise as a non-invasive nanomedicine for cataract prevention and therapy.

## Data Availability

The data presented in this study are available on request from the corresponding author.

## Supplementary Materials

The following supporting information can be downloaded at https://www.mdpi.com/article/10.3390/antiox15010118/s1: Figure S1: Characterisation diagram of Fullerenol. (A) Transmission electron microscopy image revealing the morphology of Fullerenol. The scale bar is 2 μm, and the insert image scale bar is 200 nm. (B) DLS reveals the particle size distribution of Fullerenol; Figure S2: Fullerenol toxicity testing at different concentrations. (A) Representative fluorescence images of mitochondrial membrane potential (MMP) changes detected by JC-1 staining. Red fluorescence indicates JC-1 aggregates, green fluorescence indicates JC-1 monomer. Scale bar—50 μm. (B) Representative immunofluorescence images of Ki67 (red) and DAPI (blue) staining in HLE-B3 cells across groups (Control, Fullerenol 80 μM, Fullerenol 160μM, Fullerenol 800μM). Scale bar—100 μm. (C) Quantification of JC-1 aggregate/monomer fluorescence ratio across groups (*n* = 3). (D) Quantification of Ki67-positive cells (*n* = 3); Statistical significance was determined using one-way ANOVA followed by Tukey’s post hoc test; ns: *p* > 0.05, * *p* < 0.05, ** *p* < 0.01; Figure S3: (A) Fluorescent image of TUNEL staining on paraffin sections of mouse lens. Scale bar—100 μm. (B) Statistical diagram of TUNEL staining fluorescence on paraffin sections of mouse lens (*n* = 3). (C) H&E-stained images of retinal paraffin sections from wild-type C57/6J mice following 160 μM Fullerenol instillation from Day 7 to Day 14. Statistical significance was determined using one-way ANOVA followed by Tukey’s post hoc test; ** *p* < 0.01; Table S1: The zeta-potential of C_60_(OH)_22_.
