# Supplementary material for: Fullerenol Eye Drops Mitigate UVB-Induced Cataract Progression by Inhibiting Oxidative Stress and Cellular Senescence
Source: Antioxidants (Basel). 2026 Jan 16;15(1):118. doi: 10.3390/antiox15010118 (PMC12838378; doi:10.3390/antiox15010118)
Supplement: Supplementary file 1 [file antioxidants-15-00118-s001.zip › antioxidants-4038209-supplementary.pdf]

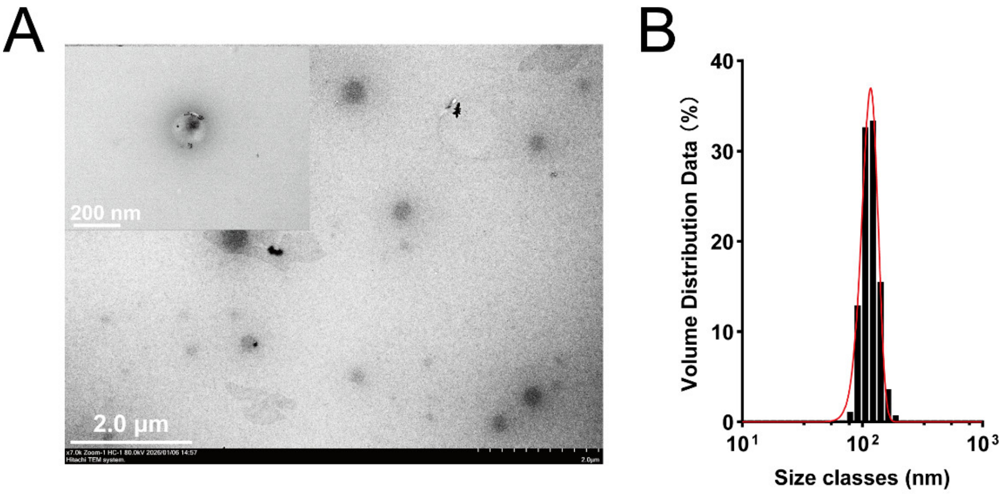

Supplementary Figure S1. Characterisation diagram of Fullerenol. (A) Transmission electron microscopy image revealing the morphology of Fullerenol. The scale bar is 2 μm, and the insert image scale bar is 200 nm. (B) DLS reveals the particle size distribution of Fullerenol.

Supplementary Table S1. The zeta-potential of $C_{60}(OH)_{22}$.

**Zeta-potential**	**Day 0**	**Day 14**
$C_{60}(OH)_{22}$	-41.2±4.61	-42.9±8.30

Zeta potential of Fullerenol（$C_{60}(OH)_{22}$）in aqueous solution stored at 4°C for 0 Day and 14 Day.

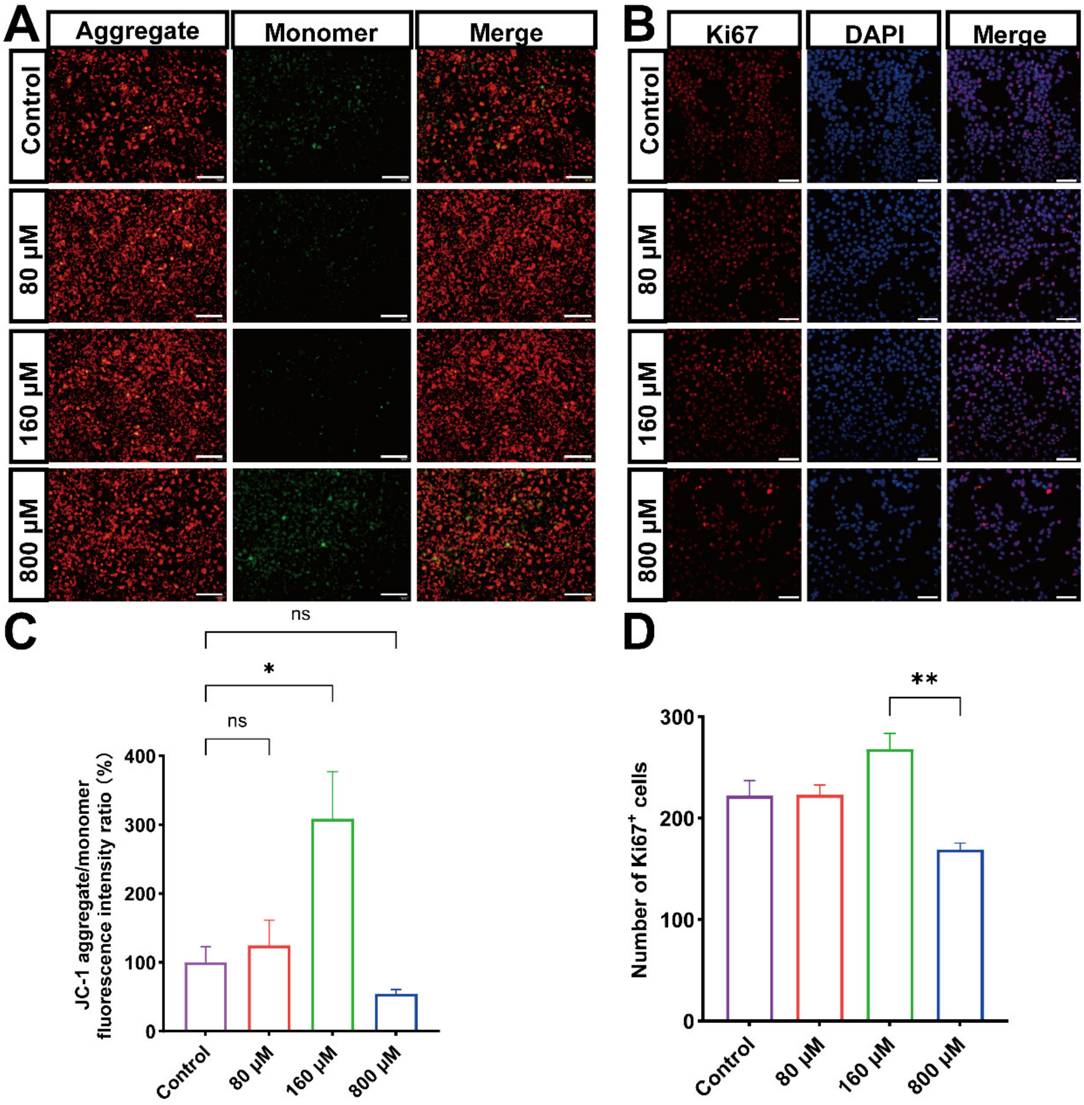

Supplementary Figure S2. Fullerenol toxicity testing at different concentrations. (A) Representative fluorescence images of mitochondrial membrane potential (MMP) changes detected by JC-1 staining. Red fluorescence indicates JC-1 aggregates, green fluorescence indicates JC-1 monomer. Scale bar — 50 μm. （B）Representative immunofluorescence images of Ki67 (red) and DAPI (blue) staining in HLE-B3 cells across groups (Control, Fullerenol 80 μM, Fullerenol 160μM, Fullerenol 800μM). Scale bar—100 μm. (C) Quantification of JC-1 aggregate/monomer fluorescence ratio across groups (n = 3). (D) Quantification of Ki67-positive cells (n = 3). Statistical significance was determined using one-way ANOVA followed by Tukey's post hoc test; ns: $p > 0.05$, * $p < 0.05$, ** $p < 0.01$.

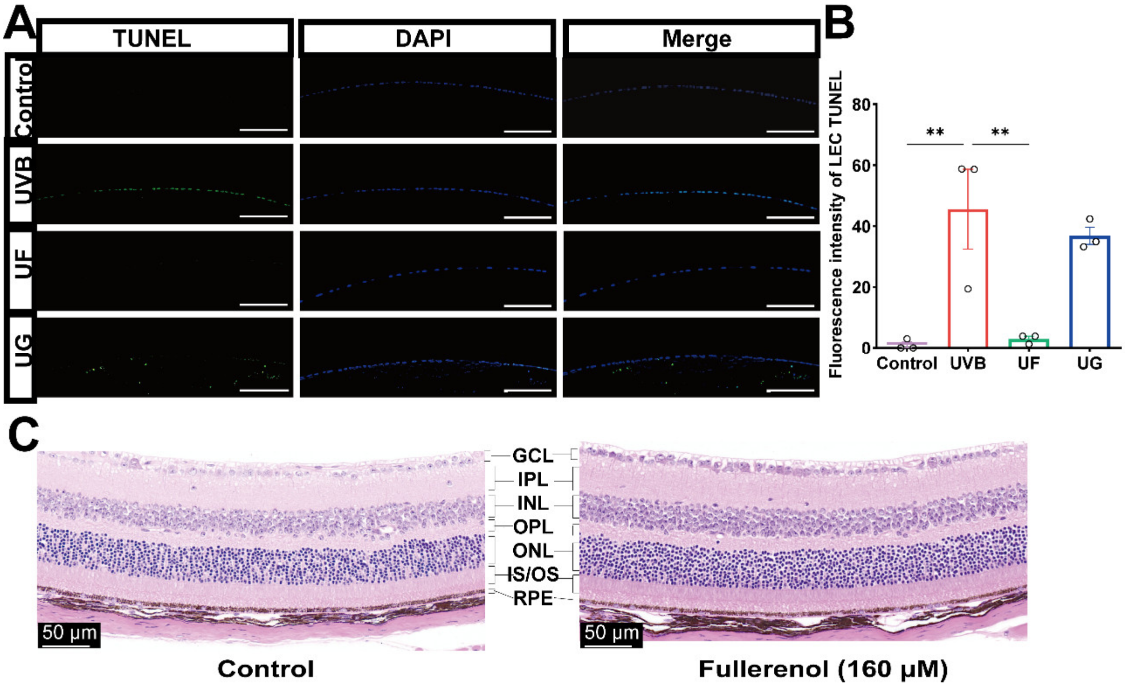

Supplementary Figure S3. (A) Fluorescent image of TUNEL staining on paraffin sections of mouse lens. Scale bar— 100 μm. (B) Statistical diagram of TUNEL staining fluorescence on paraffin sections of mouse lens (*n*=3). (C) H&E-stained images of retinal paraffin sections from wild-type C57/6J mice following 160 μM Fullerenol instillation from Day 7 to Day 14. Statistical significance was determined using one-way ANOVA followed by Tukey's post hoc test; ** $p < 0.01$.
